# Implementation of a Personalized Digital App for Pediatric Preanesthesia Evaluation and Education: Ongoing Usability Analysis and Dynamic Improvement Scheme

**DOI:** 10.2196/34129

**Published:** 2022-05-05

**Authors:** Yaron Connelly, Roni Lotan, Yitzhak Brzezinski Sinai, Dan Rolls, Amir Beker, Eilone Abensour, Orit Neudorfer, Daniel Stocki

**Affiliations:** 1 GistMD Tel Aviv Israel; 2 The Israeli Center for Emerging Technologies in Healthcare Samir Medical Center Zerifin Israel; 3 Department of Anesthesiology and Intensive Care Tel Aviv Medical Center Tel Aviv Israel; 4 Sackler Faculty of Medicine Tel Aviv University Tel Aviv Israel; 5 Faculté de Pharmacie Université de Paris Paris France; 6 Dizengoff Pediatric Community Center Clalit Health Services Tel Aviv Israel

**Keywords:** mHealth apps, preanesthesia, pediatric setting, mHealth usability, usability analysis, mobile health, mHealth, pediatrics, anesthesia

## Abstract

**Background:**

Preanesthesia evaluation is a basic practice preceding any surgical procedure, aimed at tailoring individualized anesthetic plans for patients, improving safety, and providing patients with educational knowledge and tools in preparation for the surgery day. In the last 2 decades, eHealth and mobile health (mHealth) settings have gradually replaced part of the face-to-face encounters as the platform for preanesthesia communication between doctor and patient, yielding a range of benefits as demonstrated in recent publications. Nevertheless, there is a lack of studies examining the effectiveness of surgical mHealth apps focusing on the pediatric preanesthetic setting and addressing their usability among families.

**Objective:**

This study describes a dynamic approach for the development process of GistMD’s preanesthesia mHealth system, a mobile-based educational and management system designed for the pediatric setting.

**Methods:**

The study was conducted in 4 departments at a 1500-bed quaternary, academic medical center in Tel Aviv, Israel. During the study period, the link to the preanesthesia system was sent via SMS text messages to families whose children were about to undergo surgery. The system included preanesthesia questionnaires, educational videos, downloadable instructions, and consent forms. Continuous collection and examination of usability data were conducted during the implementation term including responsiveness, effectiveness, and satisfaction indicators. The information collected in each stage was used to draw conclusions regarding potential usability gaps of the system and to plan product adjustments for the following period.

**Results:**

During 141 days of implementation, the link to the GistMD preanesthesia management system was sent to 769 families, and product-fit actions were implemented during this term: (1) changing text message scheduling for addressing learnability and accessibility, resulting in a significant increase of 27% (*χ*^2^_1_=12.65, *P*<.001) in view rates and 27.4% (*χ*^2^_1_=30.01, *P*<.001) in satisfaction rates; (2) reducing the number of screens to increase efficiency and operability, leading to a significant decrease of 8.6% in cases where users did not perform any activity on the system after logging in (*χ*^2^_1_=6.18, *P*=.02); (3) conducting a patient-focused campaign in 2 departments aimed at addressing memorability, leading to significant increases in 8 of the 12 usability indicators.

**Conclusions:**

Our results indicate that mHealth product-fit decisions originating from theory-based approaches and ongoing usability data analysis allow tailoring of the most appropriate responses for usability gaps, as reflected in increased use rates and satisfaction. In the case of the preanesthesia management system in the pediatric setting, increased usability conveyed important benefits for patients and families. This work suggests a framework and study methods that may also be applicable in other mHealth settings and domains.

## Introduction

Every surgical procedure that involves anesthesia is preceded by a preanesthesia evaluation session, conducted to develop a plan for anesthesia and assess the potential risks. Generally, it includes 2 essential components. First, to tailor an anesthetic plan for each patient, anesthesiologists conduct the preanesthesia clinical evaluation by gathering information from medical records, physical examinations, and patient interviews [1-3]. This basic practice aims to improve patient safety; in addition, it is important for health care organizations, as it can minimize preoperative costs, operation cancellations, and operating room time loss resulting from managing suboptimal plans [4]. Second, the process is also designed to provide patients with educational knowledge and tools that will enable them to be better prepared for the surgery day and more engaged in the anesthesia process they are about to undergo, facilitating shared decision-making and reducing preoperative anxiety, of which anesthesia is a leading cause [5-10]. The preanesthesia process is particularly important in the pediatric setting as children's anesthesia carries the risk of unique complications, such as respiratory adverse events and involves emotional engagement of the child and its parents, who usually experience significant anxiety and seek to be informed by care providers [11,12].

In the last 2 decades, telemedicine, eHealth and mobile health (mHealth) settings have gradually replaced part of the face-to-face encounters as the platform for preanesthesia communication between doctor and patient [13], and a considerable amount of literature demonstrated the range of benefits resulting from that shift. For example, a study conducted at 4 veteran affairs medical centers in the United States revealed that the electronic consultation system allowing primary care providers and anesthesiologists access to a shared electronic record can improve workflows of anesthesiologists and allow the development of anesthetic plans without face-to-face preoperative visits [14]. Another study conducted in the United States demonstrated that telemedicine screening visits, prior to preadmission testing center appointments, decreased the time spent in hospital on the preoperative day, prevented case cancellations, and highly increased patient experience and satisfaction [15]. Nonetheless, one of the most important values that is addressed using these technologies from a patient perspective is the access to quality care. For instance, a study in Canada, where nearly 15% of the residents live in remote areas, showed that 9 out of 10 patients that live far from the hospital and 8 out of 10 anesthesiologists were highly satisfied with the experience of using telemedicine-based preanesthesia assessment [16]. In fact, similar results were demonstrated among patients from a central area with shorter travel distances and expenses [17]. The vital role of telemedicine capabilities was well demonstrated during the COVID-19 pandemic [18,19], highlighting the need to keep developing innovative solutions that will provide remote access for the preanesthesia domain, regardless of distance-from-hospital considerations.

Although worldwide smartphone penetration reached 48.3% in 2021 [20] and more than 400,000 mHealth apps are available in app stores [21], mHealth apps in the wide context of surgical and operative settings are increasingly emerging and offer new opportunities for patients [22,23]. The efficacy of these apps was recently measured in several studies that underline the potential role of mHealth platforms in promoting patients’ comprehension and readiness prior to surgery, adherence to proper postoperative behavior and patient outcomes [23]. A South Korean study presented a significant increase in patient knowledge regarding surgical safety issues before the operation when using guidance apps [24]. In another study, anxiety and depression scores among patients with breast cancer were significantly lower when they accessed additional information provided by an mHealth app [25]. Postoperative behavior was examined in patients who underwent bariatric surgery and demonstrated a greater tendency to adopt a healthy lifestyle because of using tailored educational apps [26]. Despite the unique need, only a few studies have examined the effectiveness of surgical mHealth apps focusing on the pediatric preanesthetic setting and were designed for parents and their children. Some of these studies have found that preoperative anesthesia education provided by mobile apps can reduce anxiety and improve patient satisfaction [27,28], but, to the best of our knowledge, none of the studies addressed their usability among parents and children. In contrast, there are several examples of usability studies that are intended for health care providers, professional anesthesiologists, facilitating information sharing and management [29-32], or postoperative settings [33]. Leveraging the benefits of mHealth requires families to be highly engaged in using the apps [34]. It is evident that an incredibly low portion of the 400,000 health care apps has been successfully implemented; the total number of worldwide downloads sharply decreased recently, and hospitals can engage only 2% of their patients in mHealth activities [21]. This environment highlights the importance of evaluating mHealth apps via the prism of usability, assuming that one of the reasons that considerable proportions of users, patients, or professionals may not use the apps owing to low-quality or poor user interface (UI) or user experience (UX) components and not necessarily because of their low potential value [34]. The acceptable basic usability characteristics of systems and software products emerged in 1990 and are based on the definition of the International Organization for Standardization that encompasses effectiveness, efficiency, and satisfaction [35,36]. The Nielsen model expanded the conceptualization of usability and included learnability, memorability, and error protection [37]. The 6 main themes of usability should reflect a variety of subcharacteristics such as UI aesthetics, defined as the degree to which a product or system has attributes that make it easy to operate and control, and the degree to which a product or system can be used by people with the widest range of characteristics [36].

Considering the lack of literature evaluating the usability of mHealth products that are designed for patients and focused on the preanesthesia period in a pediatric setting, this study aims to describe the implementation process of a mobile-based system designed for this purpose.

## Methods

### Study Objectives

The main objective of the study is to report a dynamic, quick, and timely approach for mHealth product development during its implementation period applied to modify the web-based app to family needs and address usability gaps as quickly as possible. This agile process included ongoing collection of usability data from day 1 and theory-grounded thinking. The secondary aim is to describe the lessons learned and suggest recommendations to increase the usability of these types of systems.

### Web-Based App

The mHealth system used in this study was developed through a partnership between an industry vendor (GistMD, Ltd) and a 1500-bed general academic medical center in Israel (Tel Aviv Sourasky Medical Center). The medical center is the largest acute care facility in Israel. GistMD is a digital health company founded in 2018 and develops platforms that enable scalable production of personalized content and media for improving patient education, engagement, and adherence.

The system was designed to address three main functions in the pediatric preanesthsesia meeting: (1) providing families and children with personalized education regarding the anesthesia process and the required preparations prior to surgery, (2) performing a remote preanesthesia evaluation possibly avoiding the need for a face-to-face encounter prior to the day of surgery, and (3) giving families the opportunity to download instructions, consent forms, and other relevant information prior to the day of surgery.

**Figure 1:**
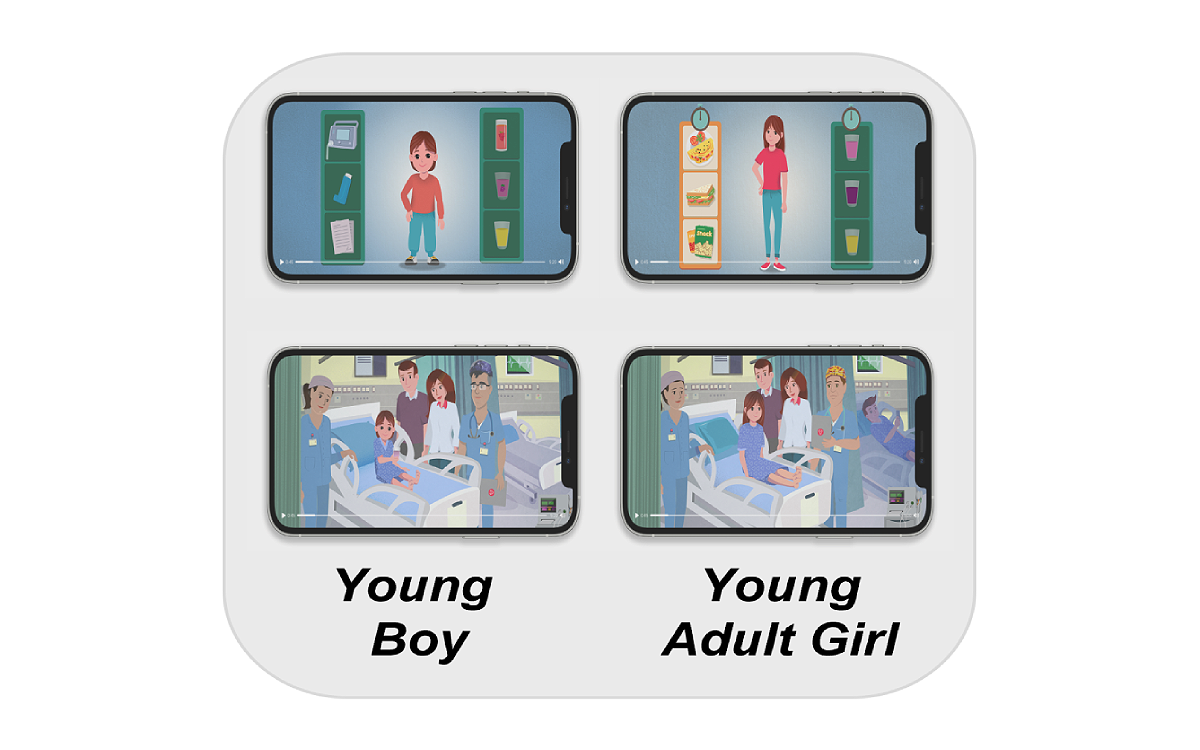
Illustration of various combinations of visual content.

The process includes the following steps. A text message is sent to the families’ mobile phones in conjunction with scheduling the surgery for the children and entering minimal demographic data into the system (gender and age group 0-8 or 9-18 years). The message is sent from the medical center and contains a hyperlink for the web-based system. The web-based app is compatible with all types of smartphones. Initially, the guardians are asked to indicate their preferred language for all the content in the app. Currently available languages are Hebrew, English, Arabic, Russian, and Spanish. The app customizes the content according to the medical condition for which the child was referred for surgery and demographics (eg, language, age, gender, and background diseases), displaying highly personalized material. In total, the system’s recombination engine can produce 80 different versions of the video, whereas the families can reboot the system at any stage and dynamically adjust the preferences or customize a new version of the video (eg, when different users want to watch the video in different languages). The guardians were also requested to answer a 10-item preanesthesia evaluation questionnaire aimed at identifying patients with a medical background requiring face-to-face evaluation by an anesthesiologist prior to the day of surgery. The questionnaire is an in-house development of the Tel Aviv Medical Center, validated among 200 patients for multilingual versions since 2019. It has become a standard of care at the medical center since the middle of 2020. Immediately after filling out the questionnaire, the family has the option to watch a tailored 5-minute animated video aimed at assisting them to better prepare for the day of surgery. After watching the video, parents can download various documents to review, including the informed consent for anesthesia, written instructions for fasting guidelines before anesthesia, a checklist of documents to bring on the day of surgery, and a list of new onset symptoms that require attention prior to surgery. The family is requested to provide feedback by stating whether the video was helpful or not. As illustrated in Figure 1, the characters and the storyline in the video, as well as the documents, are adjusted according to the combination of demographics entered.

### Study Population

Between August 9, 2020, and December 31, 2020, the app was offered to all families whose children were scheduled for surgery in 4 pediatric surgical departments at Dana-Dwek Children’s Hospital, the pediatric facility of the Tel Aviv Sourasky Medical Center in Israel. The medical center serves a population of 1 million people from Tel Aviv and the greater metropolitan area, a central location that is typically characterized by higher socioeconomic levels. Dana-Dwek Children’s Hospital provides medical services and treatment for children of all ages, from newborns to 18 years of age and approximately 3500 pediatric surgeries are performed at the hospital annually. The 4 departments selected for the study represent approximately 80% of the surgical capacity of the pediatric hospital in the study term and specialize in plastic surgery, urology, orthopedics, and otolaryngology.

### Data Collection

Surgery coordinators in the 4 pediatric surgical departments were equipped with an operation interface that supports the web-based app. The operation interface and the web-based app are cloud-based and external to the hospital’s information technology infrastructure. After the coordinator scheduled a surgery and the system sent a text message to the guardians of the child, usability data were automatically retrieved for each user without the need for intervention or active collection among families. The collected data were encoded and anonymized, and the personal details of the patients or their family could not be identified.

### Ethical Considerations

This manuscript is not considered human subjects research and is not presenting any medical information, and thus does not require ethical approval.

### Measurements

Usability indicators measured in this study were focused on the degree of usage of the various functions of the app. All measures were coded as dichotomous variables, with 1 indicating that the users used the function and 0 indicating that they did not. At the basic level, the initial indicator is the responsiveness to logging on to the system, showing whether the users open the link they received in the text message. Similar threshold indicators are common in similar usability tests of apps [34]. At the second level, effectiveness indicators examined whether the users used each further function (filled out and completed the questionnaire, played the video, downloaded the informed consent and the documents with instructions). Additional indicators refer to determining whether users meaningfully and efficiently used the educational function. Previous studies have measured the usability of educational content in health apps by assessing the extent of use, such as the number of modules completed or the average viewing time [26,31]. In addition to the measurement that examined whether the users clicked “play” and started watching the video, meaningful use of the educational function was measured in this study by examining whether the participants viewed 75% or more of the video’s total duration. The first 75% of the video was chosen as the cutoff point, as it contains the essential content to be provided to the families, whereas the remaining 25% contains, for example, wishes from the medical staff and credit roll. In this regard, reverse indicators examining underuse of the system were also measured by examining whether the participants viewed 50% or less of the video’s total duration and whether the users accessed the link but did not follow on with any activity. Finally, the satisfaction indicator refers to the user perspective regarding the opportunities that the system suggests. Unlike satisfaction that corresponds to the degree of enjoyability of apps [38], the measure used in this study examined whether it was appropriate for the needs of the participants and was perceived as providing them utility [36,39]. In this study, satisfaction was measured by asking participants, “Do you find the system helpful?” The response options were “Yes” and “No.” Answering the question was not mandatory and participants could use all functions without providing an answer.

### Procedures

For a quick product-fit process, we applied dynamic and ongoing processes of usability assessment. First, the implementation process for the web-based app was divided into 4 periods, and toward the end of each period, an examination of the usability indicators was conducted. All data were collected automatically, and the information gathered in each stage was used to draw hypotheses regarding potential current usability gaps of the system. Next, up-to-date literature and theories on the usability of software applications and mHealth in particular were reviewed to tailor product adjustments for addressing the gaps in the following period. Table 1 describes the thinking processes that yielded the product-fit strategies and actions taken to address the usability gaps during the market-product fit process in all stages of implementation.

**Table 1:** Product-fit actions by strategies and related usability gaps.

Period	Cases (N)	Duration (days)	Usability gap	Decision on strategy	Decision on action
1: Launching	286	34	Poor usage rates of basic features	Addressing learnability and accessibility issues by considering the daily habits of end users	*Change in text message scheduling*
2	56	22	Unsatisfied rates of underuse tendency	Improving efficiency and operability by applying lean and agile thinking for product modification	*Reduce number of screens*
3	238	54	Usability gaps between different departments	Addressing memorability problems by sending notifications	*Family- focused campaign*
4: End of implementation	189	31	N/A^a^	N/A	*Follow-up and summarizing*

^a^N/A: not applicable.

#### Period 1: Addressing Learnability and Accessibility

##### Identify the Gap

The web-based system was launched on August 9, 2020. At 34 days after the initial distribution, we conducted a preliminary evaluation of its usability indicators. The findings (as specified in the results section below) revealed that although users opened the link for the system and filled out the questionnaire, the usage rates of users who played the video and started watching (33%) and downloading the documents (1%) were extremely low. These poor results led us to conduct a deeper viewing analysis suggesting that although users get the link for the system in the morning (as was the custom at first), more than a third of them preferred to watch the video in the afternoon, and those who watched the video in morning hours tended to watch shorter parts of it.

##### Choose a Strategy and Tailor the Solution

In the context of usability, learnability should reflect the degree to which the system can be used to achieve the desired goals of learning and reap its benefits. Learnability can be facilitated by integrating the system with the daily life and habits of users, while accounting for the amount of time required for users to perform the process [35,37]; this type of modification can also address the accessibility aspect of usability while allowing more users with the widest range of characteristics and capabilities to use the system [36]. Identifying the gap between the time when users receive the link to the system and their preferred hours to use it in practice led us to conclude that the required step is to change the hour when the link is sent to users from morning to afternoon, when users are more likely to use the feature and invest more effort for the time-consuming activity of watching the video.

#### Period 2: Addressing Efficiency and Operability

##### Identify the Gap

The second period occurred between September 14 and October 6, 2020, and it yielded 56 cases. Toward the end of the period, we re-evaluated the usability indicators and analyzed the underuse trends. As specified in the results section below, although all the usability indicators increased, particularly the rate at which families that opened the link increasing by 10%, we expected that the rate at which users logged into the system by opening the link without any activity would decrease in relation to the high value it showed in the first period, but it remained stable with a high and dissatisfying rate of 11%. The increase in the rate of system logins, alongside the high rate of underuse, led us to hypothesize that users may want to use the system but find it cumbersome and it may be possible to improve UX.

##### Choose a Strategy and Tailor the Solution

When it comes to software products, the efficiency aspect of usability can be attributed to the stability of the software as well as the quality and aesthetics of the UI that make the product easy to operate and control [35,36]; therefore, user-centered interface designing is strongly related to usability [40,41].

**Figure 2:**
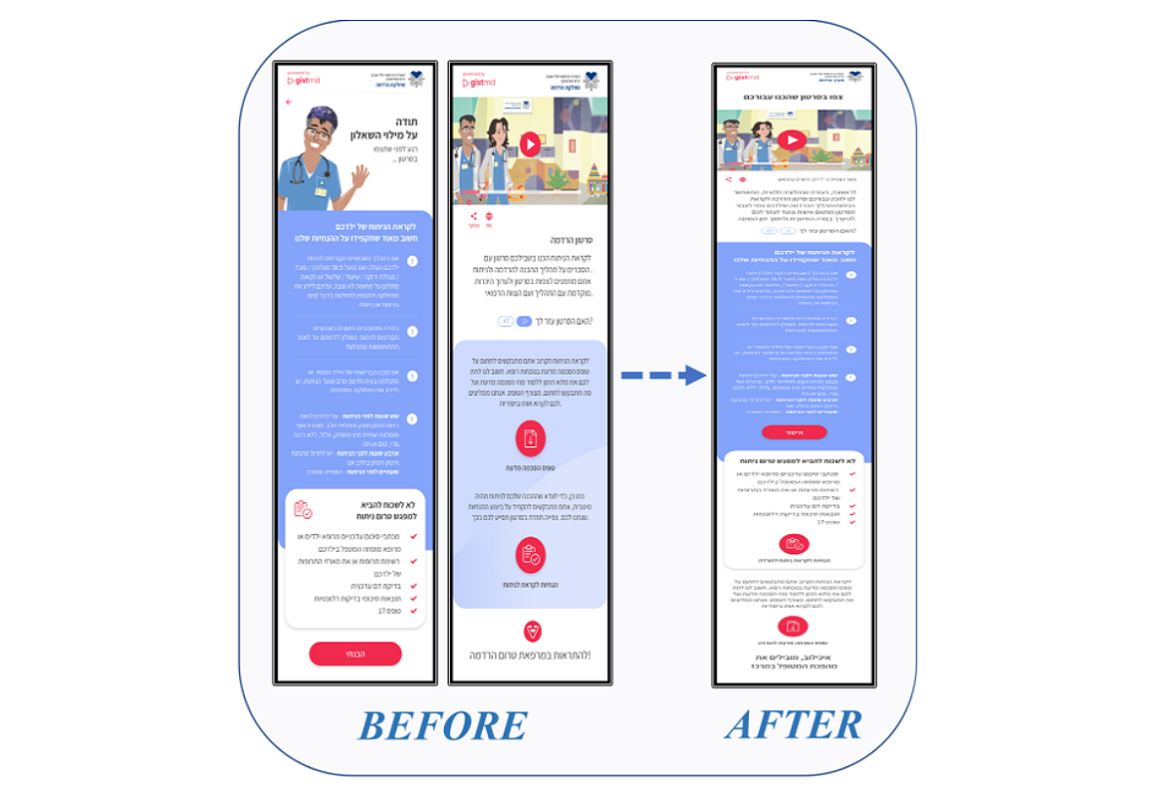
Illustration of the differences between the 2-screen and 1-screen versions of the app.

A key strategy for improving usability by simplifying the UX relies on “agile” and “lean” thinking of designing software products [41,42] and mHealth apps in particular [35,43]. Agile and lean design underlines the need to produce high customer value while minimizing the elements that do not provide value for providers or users. From these perspectives, individual functions are implemented in the smallest possible steps [41,44,45]. The solution chosen to mitigate the underuse tendency was reducing one of the screens [46]. Initially, instructions for the family prior to surgery were displayed on a screen separate from the video player, and the download features of the informed consent and the printable detailed instructions. Users were required to click on a button to move from the first screen to the second. After the modification, all functions were merged into 1 screen, allowing a more compact app whose flow of use is shorter and simpler. All features were accessible to users by just scrolling down. Figure 2 presents screenshots from the app that illustrate the differences between the full version and the simplified one.

#### Period 3: Addressing Memorability

##### Identify the Gap

After the measures aimed to mitigate the tendency to underuse the system, the third period of implementation took place between October 7 and November 30, 2020, and this yielded 238 cases. In this stage, usability indicators were examined across different departments in the first 3 periods. As specified in the results section, the system performance in 2 departments (C and D) was significantly lower compared to that in the other 2 departments (A and B).

##### Choose a Strategy and Tailor the Solution

**Figure 3:**
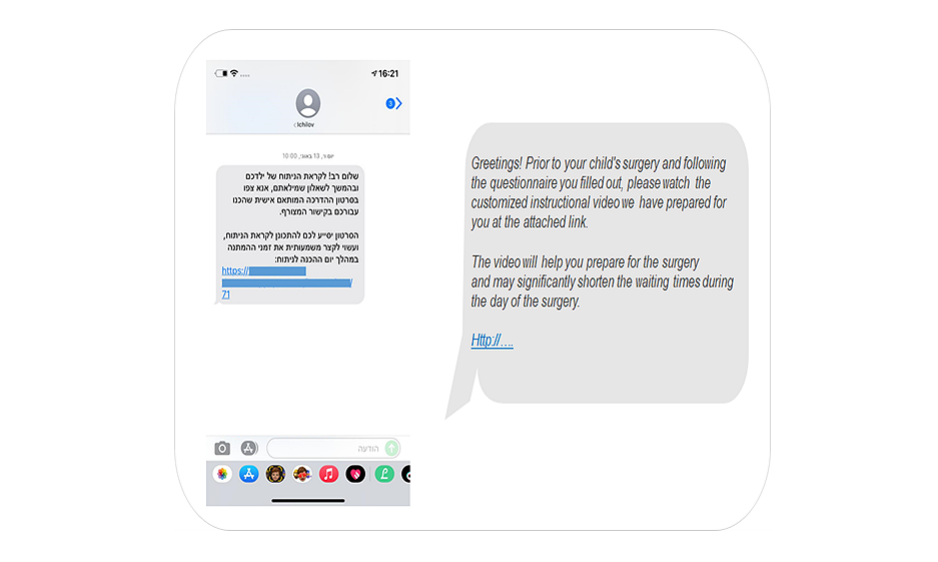
Illustration of a notification sent to families as part of the campaign.

Memorability refers to the level of ease with which users, families in this case, can recall how to use an app even after discontinuing its use for some time [47]. Effective notification management is important for maintaining memorability of mobile apps among users and consequently increasing the usability potential [48]. In practice, departments C and D were targeted in a focused campaign aimed to strengthen memorability and thus increase their usability performance and reduce the gaps between them and departments A and B. Families whose children are about to undergo surgery received telephone and SMS text message notifications regarding the opportunity to use the web-based system and the benefits of using it. Figure 3 illustrates a screenshot of the message.

#### Period 4: Follow-up and Examining Trends Throughout the Implementation Term

At the end of the implementation term, usability indicators across departments were compared to assess the impact of product-fit actions applied in the third period. In addition, trends related to all usability indicators and with respect to the entire sample were examined. The final implementation period was between December 1 and 31, 2020, and it yielded 189 cases.

### Statistical Analysis

SPSS software (version 26; IBM Corp) was used to perform data analysis. To examine the effect of the strategic actions of product fit, chi-square tests were performed for comparing all usability indicators (dichotomous variables) during each of the periods in comparison to the prior periods. The percentage of users who opened the link was calculated considering all participants to whom a message was sent. The percentage of users with respect to all other usability metrics was calculated only from the total number of users who opened the link.

## Results

### Sample

During the 141 days of the implementation term, the GistMD preanesthesia system was delivered to 769 families of children who were about to undergo surgery at the Tel Aviv Sourasky Medical Center. The system was initially launched in 4 departments. Table 2 presents the characteristics of the pediatric patients whose families received the link to the system prior to their surgery from the various departments.

**Table 2:** Patient characteristics by department (N=769).

Characteristic			Department A		Department B		Department C		Department D		Total
**Age in years, n (%)**											
	0-8	66 (67.3)		145 (82.9)		158 (66.7)		70 (27)		439 (57.1)	
	9-18	32 (32.7)		30 (17.1)		79 (33.3)		189 (73)		330 (42.9)	
**Gender, n (%)**											
	Male	67 (68.4)		154 (88)		152 (64.1)		133 (51.4)		506 (65.8)	
	Female	31 (31.6)		21 (12)		85 (35.9)		126 (48.6)		263 (34.2)	

### Step 1: Change in Text Message Scheduling

The launch period was between August 9 and September 13, 2020, yielding a total of 286 cases. The initial findings indicate that 227 (79.4%) families received and opened the link provided by the system, 195 (85.9%) opened the link and responded to the questionnaire, which is the first step in the system’s operational flow; however, only 75 (33%) of them played the video and started watching it. Moreover, only 3 (1%) and 4 (1%) families downloaded the informed consent form and the instructions document, respectively. Finally, the number of users who answered “YES” to the question *“*Was the system useful to you?” corresponded to only 7% (15/227) of the families who received and opened the link.

The second period was between September 14 and October 6, 2020, and it included 56 families who received the link, with only 89% (n=50) opening it. Chi-square tests showed marginal significance indicating a 9.9% increase in the second period compared to the first one (*χ*^2^_1_=2.99, *P*=.06). However, strong statistical significance was observed for the other usability indicators. First, 30 (60%) of the parents opened the link and played the video, which is an increase of 27% in comparison to the first period (*χ*^2^_1_=12.65, *P*<.001). Second, the proportion of parents who answered the questionnaire but did not watch the video decreased from 52.9% (120/227) in the launching period to 28% (14/50) in the second period (*χ*^2^_1_=10.14, *P*=.001). Third, the download rates of informed consent forms and instruction documents increased by 18.7% (*χ*^2^_1_=31.96, *P*<.001) and 20.2% (*χ*^2^_1_=32.76, *P*<.001), respectively. Finally, 34% (17/50) of the families actively indicated that the system was helpful for them, an increase of 27.4% in comparison to the first period (*χ*^2^_1_=30.01, *P*<.001).

### Step 2: Reduce Number of Screens

The third implementation period was between October 7 and November 30, 2020, and it included 238 cases. Results indicate that the families who opened the link without any activity in the system significantly decreased from 12% (6/50) in the second period to 3.4% (7/238) in the third (*χ*^2^_1_=6.18, *P*=.02). The number of cases in which the video was stopped before reaching its midpoint also decreased from 20% (6/30) to 8.6% (12/140). Chi-square tests demonstrated a marginal significance (*χ*^2^_1_=3.41, *P*=.07). It is noteworthy that all the values of the usability parameters, which increased in the second period, were retained or increased in the third, but without significant differences; for instance, the satisfaction indicator rose from 34% (17/50) in the second period to 42% (88/206) in the third period (*χ*^2^_1_=0.86, *P*=.17).

### Step 3: Patient-Focused Campaign

The fourth period was form December 1 to 31, 2020, in which 124 cases were retrieved and analyzed. Table 3 presents usability comparisons across different departments in the first 3 periods. The system performance in 2 departments (C and D) was lower than that in the others (A and B). For example, the link opening rate in department A was 90.8% (59/65), whereas that in department D was only 71% (142/200) (*χ*^2^_1_=10.47, *P*<.001). In addition, the difference in the informed consent download rate between department B (26/129, 20.2%) and department C (13/153, 8.5%) is also significant (χ^2^_1_=8.12, *P*=.005).

Considering these gaps, departments C and D were targeted in a focused campaign, and a follow-up examination was performed in period 4. As observed in Table 3, the campaign led to a significant increase in some of the usability indices in departments C and D. However, during the campaign, some of the usability indicators of departments A and B, which were not part of the campaign, decreased, except for 1 (download of informed consent document); the decreases were not significant.

**Table 3:** Usability indicators before and after the focused campaign across departments.^a^

Department				Period 1 (N=238)		Period 2 (N=56)		Period 3 (N=238)		Periods 1 to 3 (N=580)		Period 4 (N=189)		*χ*^2^ (*df*)		*P* value
**Department A (not targeted during the campaign)**				n=8		n=13		n=44		n=65		n=33				
	Opened the link (%)		88		92		91		91		100		*3.25 (1)*		*.08*	
	Responded to the evaluation questionnaire (%)		100		67		98		92		91		0.01 (1)		.6	
		Played the video and started watching (%)	71		58		80		75		73		0.38 (1)		.52	
		Watched more than 75% of the video (%)	57		58		68		64		52		1.46 (1)		.16	
		Downloaded informed consent form (%)	0		17		35		27		9		*4.2 (1)*		*.03*	
		Downloaded instructions document (%)	0		25		23		20		5		0.38 (1)		.38	
		Indicated that the app is helpful (%)	0		25		45		36		36		0.001 (1)		.56	
**Department B (not targeted during the campaign)**				n=76		n=11		n=56		n=143		n=32				
		Opened the link (%)	86		91		96		90.2		91		0.005 (1)		.62	
		Responded to the evaluation questionnaire (%)	88		100		98		93		100		2.14 (1)		.15	
		Played the video and started watching (%)	31		80		69		50.4		66		2.17 (1)		.1	
		Watched more than 75% of the video (%)	23		60		61		41.9		48		0.4 (1)		.34	
		Downloaded informed consent form (%)	3		40		37		20.2		24		0.23 (1)		.4	
		Downloaded instructions document (%)	3		40		33		18.6		17		0.03 (1)		.55	
		Indicated that the app is helpful (%)	8		40		44		25.6		41		*2.9 (1)*		*.07*	
**Department C (targeted during the campaign)**				n=77		n=25		n=70		n=172		n=65				
		Opened the link (%)	87		88		91		89		91		0.17 (1)		.44	
		Responded to the evaluation questionnaire (%)	78		96		98		88.9		100		*7.27 (1)*		*.003*	
		Played the video and started watching (%)	16		50		70		43.8		81		*24.21 (1)*		*<.001*	
		Watched more than 75% of the video (%)	13		32		63		36.6		66		*14.99 (1)*		*<.001*	
		Downloaded informed consent form (%)	0		14		16		8.5		36		*23.22 (1)*		*<.001*	
		Downloaded instructions document (%)	0		14		14		7.8		34		*22.56 (1)*		*<.001*	
		Indicated that the app is helpful (%)	8		32		42		25.5		64		*27.88 (1)*		*<.001*	
**Department D (targeted during the campaign)**				n=125		n=7		n=68		n=200		n=59				
		Opened the link (%)	70		86		71		71		83		*3.42*		*.04*	
		Responded to the evaluation questionnaire (%)	90		83		92		90.1		96		1.58 (1)		.17	
		Played the video and started watching (%)	44		67		54		48.6		61		*2.33 (1)*		*.08*	
		Watched more than 75% of the video (%)	34		50		44		38		55		*4.35 (1)*		*.03*	
		Downloaded informed consent form (%)	1		17		21		8.5		8		0.004 (1)		.61	
		Downloaded instructions document (%)	2		17		21		9.2		10		0.05 (1)		.51	
		Indicated that the app is helpful (%)	6		50		40		19		43		*11 (1)*		*<.001*	

^a^The italicized values are statistically significant.

### Step 4: Summarizing the Implementation Phase

**Figure 4:**
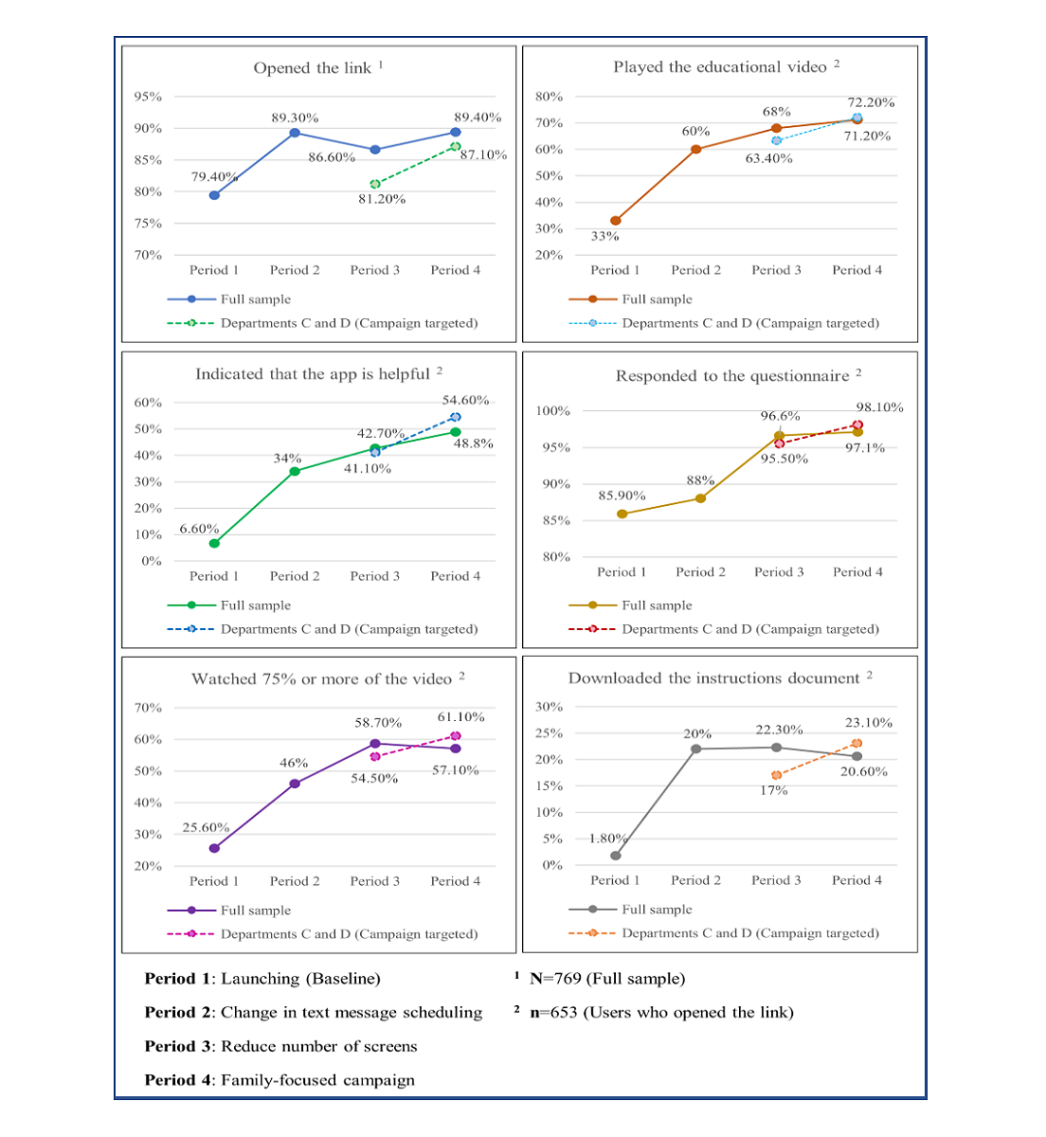
Trends in usability indicators throughout all the implementation stages.

As observed in Figure 4, analysis of the usability data for the entire sample and throughout all the stages of implementation reveals that 4 of the indices peaked in the fourth period (ie, opening the link, responding to the questionnaire, watching the video, and the satisfaction indicator). However, 3 of the indices peaked in the third period.

They demonstrated a slight decrease in the fourth period (ie, watching more than 75% of the video, responding to the questionnaire, and downloading the instruction document). Further examination of the trends only among the departments targeted during the campaign (C and D) showed that all indicators demonstrated an increase between periods 3 and 4, including those that demonstrated a decrease at the level of the entire sample. All indicators showed overall increases during the implementation period (from period 1 in comparison to the end of period 4). Chi-square tests were performed for examining the differences between indicator values at the end of period 1 and those at the end of the implementation period (period 4), revealing that all were significant at the level of *P*<.001.

## Discussion

### Overview

eHealth and mHealth platforms are gradually replacing face-to-face encounters for preanesthesia communication and evaluation, underlining their potential to streamline the process that is crucial to ensure successful surgery. Although realizing the potential of these digital solutions depends on adequate engagement among the users (ie, patients, professionals, and caregivers), it is essential to examine their usability indicators and identify barriers that might reduce the likelihood of optimal use among those who can benefit from these products. Nevertheless, studies addressing the usability characteristics of surgical mHealth apps focusing on the pediatric preanesthetic setting and designed for parents and their children are scarce. To bridge this gap, this study examined the usability of web-based preanesthesia apps that are designed for the pediatric setting and describes a product-fit process during its implementation period. Specifically, we describe 3 product modifications performed to improve the usability of the system, based on insights that emerged from the synergy between existing user data as well as academic and professional theoretical knowledge.

### Principal Findings

Our results indicate that choosing a dynamic and theory-grounded approach to product-fit adjustments during the implementation period leads to significant increases in all the usability metrics of the system (see Figure 4). Each period of the study was characterized by a different gap that was identified and addressed with a tailored response considering the conceptual principles of usability. The first action that aimed to address learnability and accessibility by considering the daily habits of end users (ie, change in text message scheduling*)* led to higher numbers of users playing the educational video and starting to watch it as well as higher questionnaire completion rates. This finding is consistent with past studies that have shown that patients having the option to receive customized flexible text messages scheduled in their mHealth apps increased their effective usage of these apps [49], and this eventually improved their health outcomes [50]. In the context of the first action taken, existing studies also suggest that users should be directed to use the app at a time that best suits them and implies that the solution should bring together the concept of usability and data science. This broad perspective indicates that proper scheduling of push notifications for smartphones has become a major challenge these days, where users receive large amounts of various information on their mobile device that cause information overload and influence their ability to pay attention to the information that we seek to provide them [51]. The data from this study revealed that users prefer to use the system in the afternoon, which may indicate that there are families with children about to undergo surgery, and they may need time without interruptions for learning and preparing for an emotionally involved event. Alternatively, but not in contradiction, if we choose to “speak about usability,” we will ask ourselves whether the families may want to coordinate app usage at a time when they can do so together with their children to enable them to be engaged in the process.

The second action, aimed to address efficiency and operability by reducing the number of “screens,” was reflected in our collected data indicating reduced instances of app underuse. This finding suggests that interfaces failing to deliver a positive UX may be a barrier to usage for an audience of users who have expressed a willingness to use the app, passed the initial threshold, and clicked on the link. By reducing the number of screens, we tried to apply a lean approach for the app that aimed to provide better customer value using lower capacity; in fact, this approach can present the entire process demonstrated in this study in a different light. In recent years, “lean thinking” demonstrates one of the theoretical meeting points between usability and UX of apps as well as for developing software products using the agile process in which the developers initially release a Minimum Viable Product (MVP) [41]. The UX of the product designed using an agile process will be tested according to the lean approach by receiving user feedback and constantly examining the usability indices of the product [52]. After analyzing the accumulated data, a new cycle will begin, and an updated MVP will be released for users in another agile process. The dynamic process of the app development described in this study also involved cycles of product modifications derived from data analysis of its usability metrics. This iterative scheme is previously demonstrated in studies that described the development process of mHealth apps and its potential to produce a better product [53] as well as increase its usability [54].

The third action aimed to address the memorability of the app among families and was implemented in response to the data analysis we performed to examine the usability indicators in each of the departments and the findings related to 2 departments that demonstrated low usability performance in comparison to the others. The focused notification campaign was able to bring about a considerable increase in the usability metrics at the departments where we hoped to achieve improvement (C and D). Previous studies have also highlighted the importance of better connectivity between providers and users of mHealth apps; using notifications for this purpose had a positive influence on the product assessment [55] and usability [56].

Although we achieved the desired result, we also found that increases in the metrics for departments A and B were characterized by higher usability indices in the baseline assessment (periods 1 to 3); therefore, these metrics were not targeted during the campaign, where extreme and modest decreases in some of the metrics during the fourth period were observed (See Table 3). The relatively weak performance in departments A and B at period 4 affected the overall results and can explain the negative trend in the values of the 3 indicators that demonstrated a decrease in the fourth period with respect to the entire sample. Contrary to the overall negative trend, departments C and D showed an increase in all indices (see Figure 4). These findings suggest that latent usability barriers may emerge when high usability indicators are demonstrated, and this highlights the need to examine usability patterns at diverse levels of analysis, such as tuning the app according to population-specific or department-specific needs. From a different viewpoint, usability may also be improved in groups of already highly engaged users, and this can further motivate them to use the app.

### Lessons Learned

There are 2 main lessons to be learned based on our positive experience with the dynamic implementation of the web-based app. First, mHealth product-fit decisions originating from usability data analysis can be reflected in increased app use and satisfaction levels. In a system designed for the pediatric setting, increasing usability involves providing important benefits (ie, education, reducing anxiety) to patients and families who initially expressed a willingness to use the system (ie, logged in and checked its functionality) but did not use it optimally, if at all they used the system. In this study, several cycles of usability analyses were performed followed by product modifications to suit the user requirements. It is possible to link this process to the concepts of agile and lean design and development, but its main strength is different and lies in its inherent ability to facilitate theory-grounded thinking aimed at interpreting data and registering metadata. The combination of the lean design principles, data sciences theory, and conceptualization of product usability characteristics enabled modifications and successful improvements to the app. Second, we realized that satisfactory usability metrics do not necessarily indicate that product-fit modifications should not be made. In the last action, which included a campaign targeting patients from 2 departments, we did not concentrate product-fit efforts in the 2 departments where we assumed the app performance was good. From the decreased usability indices observed in these departments, we can learn that maintaining usability is an ongoing process that requires deepening of the theory-grounded thinking and efforts to refine the methods of analyzing the data retrieved from the usability patterns of our users.

### Study Strengths and Limitations

The present study has 2 main strengths. First, to the best of our knowledge, this is the first study to examine usability of surgical mHealth apps that focused on the pediatric preanesthetic setting and was designed for parents and their children. Addressing the unique needs of children and families prior to surgery is particularly important owing to the emotional involvement in the preparation for the operation and information seeking by the parents. Usability analyses of mHealth solutions are aimed at maximizing the potential of exposure and usage of these solutions among patients and families who need them the most. Second, although most studies examining the usability of apps in the surgical setting relied on small samples, this study is based on comparative examination of 769 cases, thus collecting better quality data for understanding the usability of the app among patients and their families.

The notable limitations of the study concern the sample and its potential lack of representativeness. First, the fact that the system required using smartphones excluded families without access to these devices, or those having low technological orientation or other limitations preventing them from using it in practice. In this context, the reliance on smartphones was chosen to strengthen the elements of personalization and anonymization for the children and their families, which could have weakened if we had expanded the distribution channels of the system. Second, the study was conducted in a medical center in a central metropolitan area that is characterized by high socioeconomic levels, and this may lead to sample bias based on income or education levels. Nevertheless, because no personal information such as socioeconomic status or health literacy was collected from families, we could not control these factors and preform systematic sampling. These constraints might limit the generalizability of our findings and conclusions, but it helped us obtain a broad picture of how the system was used and even yielded statistically significant findings. Finally, because this study was designed to examine a newly launched mHealth system and to apply ongoing, quick, and timely solutions for emerging usability gaps, we did not consider collecting available data because it was time-consuming. These data, such as patient testimonials, may have contributed to the understanding of system usability. Future studies may be able to leverage knowledge derived in this manner using the approach we have proposed in this study.

### Looking Toward the Future

The findings this study yielded the current version of the system, which showed stable usability performance. Therefore, future studies may be conducted with considerably less time constraints. A major aspect that should be addressed in future studies is expanding our toolbox of measurements to assess the usability of future versions of the system, including collection of qualitative feedback from patients before and during implementation of new versions, to gain deep insights about UX and the use of validated questionnaires in the field of usability after implementation. When we think about the desired growth direction of pediatric preanesthesia apps, we believe that increased usage and satisfaction levels of patients should also be accompanied by increased satisfaction levels of hospital personnel. The app that is the focus of this study has high utility for professional anesthesiologists and the hospital management as well as patients and their families, and these aspects have not been addressed here. In our further studies, we would like to examine, for example, the extent to which the system makes it possible to eliminate face-to-face meetings and how this will affect staff satisfaction with the system and the attempt to persuade patients to use the app. We assume that a dynamic, theory-grounded process like the one presented here will be developed, in which we will examine the usability of the app from the perspective of the treating team; this will be add to the knowledge gained from the patients**,** close the feedback loop, and may also enable autotuning of the system in future.

### Conclusions

Usability analysis of mHealth solutions is crucial for maximizing the potential benefits that patients can receive. In this study, we developed a successful dynamic process of usability examination and product-fit adjustments of a preanesthesia app designed for the pediatric setting. A dynamic and ongoing process is suggested for analyzing and interpreting usability data. This process includes timely data collection and using of theory-based thinking to tailor the most appropriate response for addressing usability gaps. Because every change is implemented to address a need arising from earlier usage, this framework may facilitate values such as communication, reliability, and responsiveness that in turn strengthen the appropriateness recognizability of the app from the user perspective. This framework can also potentially be applicable in other mHealth settings and domains. Future studies will be able to refine the method to apply it in different settings and expand the areas of knowledge that will use it.

## Abbreviations

mHealth: mobile health
MVP: Minimum Viable Product
UI: user interface
UX: user experience
